# New certification requirement for autopsies: few things to consider

**DOI:** 10.4322/acr.2020.229

**Published:** 2021-01-20

**Authors:** Ameer Hamza

**Affiliations:** 1 University of Kansas Medical Center, Department of Pathology, Kansas City, Kansas, USA

**Keywords:** Autopsy, Competency-Based Education, Pathology, Surgical, Internship and Residency

The American Board of Pathology (ABPath) recently announced changes to the certification requirement for autopsies. The requirement now is 30 cases, effective immediately.^1^ This change is an interim adjustment until the ABPath autopsy requirement can become competency based. This changes comes from the ABPath’s recognition that the pathology training curriculum has significantly expanded to include areas such as molecular pathology, informatics, and laboratory management, and therefore reducing the numerical requirement for some other areas of training is a reasonable adjustment; and the impact of COVID-19 pandemic on the number of autopsy cases in some training programs.

Previously, we highlighted the implications of declining number of autopsies for the Pathology residents.^2^ The question now is the impact of this new change, on the residents as well as training programs. Are 30 autopsies enough to achieve competency to sign out autopsy cases? Will the residents be interested in doing more autopsies, once they understand that their requirement is fulfilled? Can the residency programs enforce their own requirements for graduation? These are some of the questions related to this new development.

The ABPath tried to answer some of these questions upfront in the announcement. In the official announcement it was stated that

[...] using data from a survey of Pathology Residency Program Directors, ABPath determined that 30 cases are a reasonable number for most residents to achieve competence in autopsy performance. The ABPath would like to emphasize that 30 cases are the minimum numerical requirement and that some residents may require more cases to achieve competency. Also, teaching and service obligations may require residents to perform more than 30 cases”.^1^

Of course, some residents do better than others and some would be able to sign out an autopsy after 30 cases, but in author’s opinion most wouldn’t. Each autopsy is different, and it is extremely difficult to learn most of the nuances of the practice in just 30 cases.

Thirty autopsies can be far less, enough or even still too much. The numeric number is relative to a trainee’s perspective. Even with decline in autopsy numbers, quite a few programs still get enough autopsies. We discussed the level of interest, of the residents in autopsy service.^3^ ^,^ ^4^ Keeping the level of interest in mind is important as it affects the service coverage by residents. Based on rotation schedule of most programs, the residents will still likely get more autopsy cases even after completion of their ABPath requirement. They will still have to do those cases if they fall on their turn anyway, but considering the already decreased level of interest the residents show in the autopsy service, it will be interesting to see how they perform on those additional cases after fulfilling their requirement.

Finally, perhaps the residency programs can enforce their own requirements for graduation, but for that they will have to disclose this information during the recruitment process. It will be interesting to see how this affects recruitment. In author’s opinion, if the only difference in two equally rated programs is their autopsy number requirement, most residency candidates will prefer the program with least requirement.

Whereas, the new requirement will help the residents to be eligible for the board examination and will also help residency programs that are struggling to keep up their autopsy numbers, it will also impact the recruitment and quality of work.
